# mRNA Decapping and 5′-3′ Decay Contribute to the Regulation of ABA Signaling in *Arabidopsis thaliana*

**DOI:** 10.3389/fpls.2018.00312

**Published:** 2018-03-12

**Authors:** Izabela Wawer, Anna Golisz, Aleksandra Sulkowska, Dorota Kawa, Anna Kulik, Joanna Kufel

**Affiliations:** ^1^Faculty of Biology, Institute of Genetics and Biotechnology, University of Warsaw, Warsaw, Poland; ^2^Institute of Biochemistry and Biophysics Polish Academy of Sciences, Warsaw, Poland

**Keywords:** abscisic acid, decapping, mRNA decay, *Arabidopsis thaliana*, SnRK2, ABA, ABA receptors

## Abstract

Defects in RNA processing and degradation pathways often lead to developmental abnormalities, impaired hormonal signaling and altered resistance to abiotic and biotic stress. Here we report that components of the 5′-3′ mRNA decay pathway, DCP5, LSM1-7 and XRN4, contribute to a proper response to a key plant hormone abscisc acid (ABA), albeit in a different manner. Plants lacking DCP5 are more sensitive to ABA during germination, whereas *lsm1a lsm1b* and *xrn4-5* mutants are affected at the early stages of vegetative growth. In addition, we show that DCP5 and LSM1 regulate mRNA stability and act in translational repression of the main components of the early ABA signaling, PYR/PYL ABA receptors and SnRK2s protein kinases. mRNA decapping DCP and LSM1-7 complexes also appear to modulate ABA-dependent expression of stress related transcription factors from the AP2/ERF/DREB family that in turn affect the level of genes regulated by the PYL/PYR/RCAR-PP2C-SnRK2 pathway. These observations suggest that ABA signaling through PYL/PYR/RCAR receptors and SnRK2s kinases is regulated directly and indirectly by the cytoplasmic mRNA decay pathway.

## Introduction

Abscisic acid (ABA) is a plant hormone that regulates major aspects of the plant's life cycle. ABA mediates plant stress responses and many developmental programs such as seed dormancy or root growth (Finkelstein, 2013). Abiotic stress, such as drought, high salinity and cold, induces ABA accumulation followed by activation of ABA-dependent network increasing the expression of stress-responsive genes, including *RD29B* and *RD20* (Fujita et al., 2011). ABA receptors, RCAR/PYR/PYL proteins (Regulatory Component of ABA Receptor/PYRabactin resistance/PYR1-Like) through protein phosphatase 2C from the group-A (PP2C) activate Sucrose Non-Fermenting 1 (SNF1)-Related protein Kinases 2 (SnRK2s). This leads to phosphorylation of a wide range of proteins, including transcription factors (TFs), ion channels, NADPH oxidase RbohF, the anion/proton exchanger CLCa, aquaporin, SWI/SNF chromatin remodeling ATPase BRAHMA (reviewed in Yang et al., 2017) and proteins involved in RNA metabolism (Umezawa et al., 2013; Wang et al., 2013; Yan et al., 2017). In the nucleus, the key SnRK2 targets are ABA-Responsive Element Binding basic Leucine Zipper Proteins (ABFs/AREBs) transcription factors (Fujita et al., 2009). Phosphorylated ABFs/AREBs in concert with other transcriptional regulators activate transcription of ABA-responsive stress related genes (Yoshida et al., 2014). In addition to the canonical ABA pathway, ABA signaling is connected and integrated with multiple other pathways that involve other kinase families and TFs. For example, during osmotic stress, the expression of stress-responsive genes is also regulated in an ABA-independent manner via induction of transcription factors, including DREB2A and DREB2B (Dehydration-Responsive Element Binding protein), which activate stress response genes containing the DRE cis-elements (Yoshida et al., 2014). In turn, the expression of some osmotic stress response genes, such as *RD29A*, which contain both DRE and ABA-responsive elements, is interdependent (Fujita et al., 2011). DREB1A, DREB2A, and DREB2C proteins have been reported to physically interact with AREB/ABF proteins, while ABF2/AREB1 and ABF3 can bind to and activate the DREB2A promoter. These interconnections support the notion that DREBs and AREB/ABFs cooperate to control the expression of ABA-regulated genes (Lee et al., 2010; Kim et al., 2011).

Apart from the regulation at the transcriptional level, stress response can be also controlled by selective mRNA degradation and translational repression (Munchel et al., 2011; Park et al., 2012; Ravet et al., 2012). In eukaryotes, cytoplasmic mRNA turnover is initiated by poly(A) tail shortening by deadenylases, followed by the DCP1/2- and Xrn1-mediated decapping and 5′-3′ degradation or by 3′-5′ degradation carried out by the exosome. Most mRNA turnover factors are evolutionarily conserved (reviewed in Siwaszek et al., 2014). In *Arabidopsis thaliana* the 5′-3′ mRNA decay machinery consists of DCP2 decapping enzyme with its activators, DCP1, DCP5, VCS, and PAT1, and the cytoplasmic exoribonuclease XRN4 (Xu et al., 2006; Goeres et al., 2007; Xu and Chua, 2009; Roux et al., 2015). It has been proposed that DCP5 and DCP1 activate mRNA decapping by recruiting DCP2 and VCS, which is followed by mRNA degradation catalyzed by XRN4, thereby preventing the transcript from being translated (Xu and Chua, 2011). As in other eukaryotes, decapping and mRNA turnover in plants is also stimulated by the heptameric cytoplasmic complex of Sm-like (LSM) proteins - LSM1-7 (Tharun, 2008; Perea-Resa et al., 2012; Golisz et al., 2013; Wilusz and Wilusz, 2013). The LSM1-7 complex in *Arabidopsis* also interacts with mRNA decapping and decay factors (e.g., VCS, PAT1) and mutants lacking LSM proteins accumulate decapped mRNAs that partly overlap with substrates affected in *dcp2* and *xrn4* plants (Perea-Resa et al., 2012; Golisz et al., 2013). Another, nuclear complex of LSM proteins, LSM2-8, is a core component of the U6 small nuclear ribonucleoprotein (snRNP) and is involved in pre-mRNA splicing (Perea-Resa et al., 2012; Golisz et al., 2013).

Genetic and molecular analyses reveal that decapping complex is essential for plant development and proper response to water stress (Iwasaki et al., 2007; Xu and Chua, 2009, 2012; Zhang et al., 2011; Perea-Resa et al., 2012, 2016; Golisz et al., 2013; Soma et al., 2017). For example, DCP1 has been shown to associate with DCP5 to promote mRNA decapping during dehydration (Xu and Chua, 2012). The function of the decapping complex is regulated by environmental signals, which control phosphorylation of DCP1 and VCS by the MPK6 kinase and ABA-unresponsive osmotic stress-activated subclass I SnRK2s, respectively, leading to global transcriptome changes (Xu and Chua, 2012; Soma et al., 2017). Recently the LSM1-7 complex has been reported to interact with selected specific and nonspecific stress-inducible transcripts to stimulate their decapping and subsequent degradation, thereby regulating the expression of downstream stress-responsive genes and modulating Arabidopsis tolerance to freezing, drought and high salt (Perea-Resa et al., 2016). Several mutants in LSM proteins (*lsm1a lsm1b, lsm4, sad1*/*lsm5*) are hypersensitive to abscisic acid, drought and salt (Xiong et al., 2001; Zhang et al., 2011; Okamoto et al., 2016; Perea-Resa et al., 2016). In turn, XRN4 is necessary for plant thermo-tolerance and degradation of 25% of the *Arabidopsis* transcriptome during early steps of heat stress (Merret et al., 2013) and, accordingly, LSM5/SAD1 protein has been implicated in targeting aberrant transcripts after heat treatment (Okamoto et al., 2016). These findings clearly show that 5′-3′ mRNA decay contributes to abiotic stress response by reprogramming the transcriptome in response to different conditions.

ABA signaling requires major timely regulated changes in the gene expression program that most likely involve mRNA degradation. We have therefore investigated the role of the 5′-3′ mRNA decay in plant's response to ABA. To comprehensively examine this process we analyzed mutants deficient in different steps of this pathway, decapping (*dcp5-1*), decapping activation (*lsm1a lsm1b*), and exonucleolytic degradation (*xrn4-5*). We show that these mutants are hypersensitive to ABA-mediated inhibition of germination (*dcp5-1*) or root growth (*lsm1a lsm1b* and *xrn4-5*). Our observations suggest that the canonical PYL/PYR/RCAR-PP2C-SnRK2 ABA pathway is modulated directly and indirectly through targeting the core ABA signaling components by mRNA decapping and XRN4-mediated decay.

## Materials and methods

### Plant material and growth conditions

*Arabidopsis thaliana* wild-type ecotype Columbia (Col-0) and *dcp5-1* (SALK_008881), *xrn4-5* (SAIL_681_E01) and double *lsm1a lsm1b* (SALK_106536, SAIL_756_305) homozygous mutant lines were used in this study. Seeds were surface sterilized with 30% bleach/0.02% Triton-X100 solution and grown on Murashige and Skoog (MS) medium supplemented with 1% (w/v) sucrose and 0.3% phytagel, under a 16 h light/8 h dark (long-day) photoperiod, and 22°C/20°C. For sterile hydroponic culture about 100 seeds grown for 14 d in 300-mL Erlenmeyer flasks containing 100 mL of one-half Murashige and Skoog medium supplemented with 100 mg/L myo-inositol, 500 mg/L MES, 10 g/L sucrose, pH 5.7. Seedlings were treated with different ABA concentrations as indicated, harvested, quickly frozen in liquid nitrogen, and stored at −80°C.

### Germination and root growth tests

For germination tests, 40 seeds were planted in MS medium containing various concentrations of ABA (0–0.5 μM, Sigma) in 4 replicas. The average number of seeds germinated each day was calculated and used to define the Pieper's index (average time required for germination of a single seed) (Jakubowski, 2015):

$$\begin{array}{l} \text{Pieper}\prime \text{s index}=\left({\text{x}}_{1}^{*}\;{\text{s}}_{1}+{\text{x}}_{2}^{*}{\text{s}}_{2}+\ldots +{\text{x}}_{\text{n}}^{*}{\text{s}}_{\text{n}}\right)/\left({\text{s}}_{1}+{\text{s}}_{2}+\ldots +{\text{s}}_{\text{n}}\right) \end{array}$$

x - number of a day from seeds dissemination
S - number of germinated seeds in subsequent days of observation
n - last day of experiment

For sensitivity to ABA at the early stages of vegetative growth 5-day-old seedlings were transferred from MS medium to MS medium with different concentration of ABA (0–10 μM). Root length was measured relative to control conditions 4 d after the transfer, for more than 30 roots for each data point. The ABA sensitivity results were subjected to a two-way analysis of ANOVA variance followed by *t*-tests using Microsoft Excel.

### RNA methods

Total RNA was isolated from 2-week-old seedlings using Trizol reagent (Sigma) according to the manufacturer's instructions. For Northern blot analysis 15 μg of total RNA was separated in 1.1% agarose/6% formaldehyde gels and transferred to a Hybond N^+^ membrane by capillary elution. Northern blots were performed using random primed probes amplified from cDNA template with appropriate primers and radioactively labeled with DECAprimeTM II kit (Ambion) and [α-^32^P]ATP (Hartmann Analytics), or oligoribonucleotide probes radioactively labeled with PNK (Thermo Scientific) and [γ-^32^P]ATP (Hartmann Analytics). Membranes were hybridized overnight with radioactive probes in PerfectHyb buffer (Sigma), washed, analyzed with PhosphorImager Typhoon FLA 9000 (GE Healthcare) and quantified with ImageJ software (Molecular Dynamics). mRNA half-life measurement experiments were carried out as described (Souret et al., 2004). Two-week-old seedlings were transferred to flasks containing a buffer (1mM PIPES, pH 6.25, 1 mM sodium citrate, 1 mM KCl, 15 mM sucrose), and after a 30-min incubation, cordycepin (150 mg/L) was added. Total RNA samples at indicated time points were extracted using Trizol reagent and analyzed by Northern blot. For real-time RT-PCR analysis samples of total RNA (50 μg) were DNase-digested with TURBO DNA-free kit (Ambion), according to manufacturer's protocol. RNA quality was checked on Nanodrop 1000. RT of 5 μg of RNA was performed in 20-μl reaction using SuperScript III reverse transcriptase (LifeTech) and mix of random hexamers (Invitrogen) and oligo(dT) according to manufacturer's protocol. cDNA samples were diluted 9 times and used as a template in qPCR using the SYBR Green I Master (Roche) and the LightCycler 480 (Roche). The AT1G13320 gene, the expression of which does not alter after abiotic stress, was used for normalization (Czechowski et al., 2005). All presented data are derived from three biological replicas, each of which represents an average of three technical replicas. The results were subjected to a two-way analysis of ANOVA variance followed by *t*-tests. Oligonucleotides used for Northern hybridization and PCR reactions are listed in Supplementary Table 1.

### In-gel kinase assay

Frozen seedlings were ground in liquid nitrogen with mortar and pestle and sonicated three times for 20 s in the extraction buffer (20 mM Tris, pH 7.5, 2 mM EDTA, 2 mM EGTA 50 mM β-glycerophosphate, 100 μM Na_3_VO_4_, 2 mM dithiothreitol [DTT], Complete protease inhibitor cocktail Roche) using approximately 0.5 mL of the extraction buffer for each 1 μg of plant material. After sonication, the extracts were centrifuged at 18,000 rcf for 30 min at 4°C, and the supernatants were used for further studies. In-gel kinase activity assays were performed using a method described previously (Wawer et al., 2010). Briefly, protein samples were separated in 12% SDS/PAGE gels with 0.5 mg/ml histone embedded in the separating gel as a kinase substrate. After electrophoresis, SDS was removed by washing in washing buffer (25 mM Tris/HCl, pH 7.5, 5 mM sodium fluoride, 0.5 mg/ml BSA, 0.1% Triton X-100, 0.5 mM DTT and 0.1 mM sodium orthovanadate) three times each for 30 min at room temperature. Proteins were renaturated overnight in renaturing buffer (25 mM Tris/HCl, pH 7.5, 5 mM sodium fluoride, 0.1% Triton X-100, 1 mM DTT and 0.1 mM sodium orthovanadate) at 4°C with three changes of buffer. The gel was incubated for 1.5 h at room temperature in 10 ml of reaction buffer (10 mM Tris/HCl, pH 7.5, 2 mM DTT, 0.1 mM EGTA, 15 mM MgCl2 and 20 μM ATP, supplemented with 50 μCi of [γ-^32^P]ATP). Unincorporated [γ-32P]ATP was removed by extensive washing in 5% trichloroacetic acid with 1% sodium phosphate. The gels were stained with Coomassie Brilliant Blue R250, dried and exposed to autoradiography.

### Immunoblotting

Western blot analysis was performed according to a standard procedure (Wawer et al., 2010) using polyclonal antibody anti-PYR1 (AS132634, 1:10,000) and anti-SnRK2.2/3/6 (AS142783, 1:2,000) from Agrisera. Anti-rabbit (Sigma Aldrich) horseradish peroxidase-conjugated antisera were used as secondary antibodies.

### Measurement of chlorophyll content

Chlorophyll (Chl) extraction and quantification were performed using 1 cm^2^ leaf discs cut from 3-week-old plants, as described (Lichtenthaler, 1987). Briefly, Chl was extracted with 100% acetone and quantified spectrophotometrically at 662 and 645 nm. Chl content (mg per cm^2^) was calculated as sum of Chlorophyll a and b:

$$\begin{array}{l} \text{Chlorophyll a}:\text{Chla}=12.25\text{ A}662\;-\;2.79\text{ A}645\;\left(\text{μ g per ml solution}\right)\\ \text{Chlorophyll b}:\text{Chlb}=21.50\text{ A}645\;-\;5.10\text{ A}662\;\left(\text{μ g per ml solution}\right) \end{array}$$

## Results

### Lack of LSM1, DCP5 or XRN4 increases sensitivity to ABA

**Figure 1:**
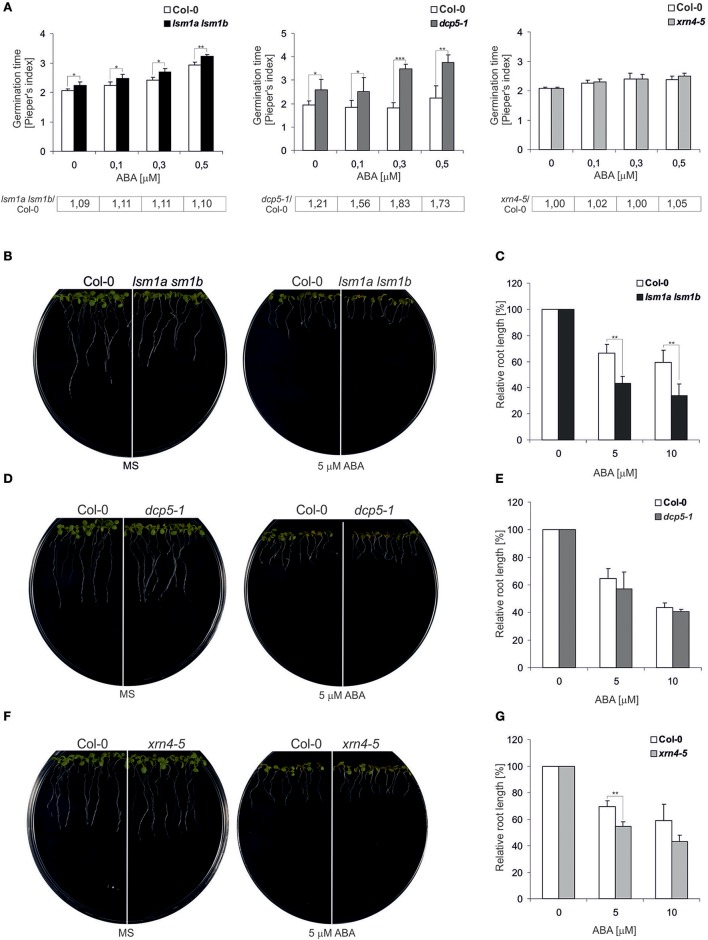
*lsm1a lsm1b, dcp5-1*, and *xrn4-5* mutant plants are sensitive to ABA. **(A)** Comparison of the average time required for the germination of a single seed of the wild-type (Col-0) and *lsm1a lsm1b, dcp5-1*, and *xrn4-5* mutants grown in MS medium containing the indicated concentrations of ABA. The numbers of seeds germinated each day were counted from at least 4 independent Petri dishes with around 40 seeds in each and Pieper's index was calculated. The ratio of Pieper's Index for each mutant vs. Col-0 in the presence of ABA is shown below each graph. Error bars represent standard deviation (SD). Asterisks indicate significant differences between Col-0 and the mutants for each ABA concentration (*n* = 4, ^*^P ≤ 0.05, ^**^P ≤ 0.01, and ^***^P ≤ 0.001). Experiments were repeated at least two times. **(B,D,F)** Root growth of Col-0 and *lsm1a lsm1b, dcp5-1*, and *xrn4-5* mutants in the presence of ABA. Five-day-old seedlings were transferred from MS medium to MS medium containing 5μM ABA. Pictures, representing one of three replicates, were taken 4 days after seedlings transfer to ABA. **(C,E,G)** Quantification of Col-0, *lsm1a lsm1b, dcp5-1*, and *xrn4-5* mutants ABA induced root growth inhibition. Five-day-old seedlings were transferred from MS medium to MS medium containing the indicated concentrations of ABA. Root length was measured relative to control conditions 4 d following the transfer. More than 30 roots were measured for each data point. Data represent means of three independent experiments. In each of the concentration of ABA were 3 independent Petri dishes. Error bars indicate SD (*n* = 3, ^*^P ≤ 0.05, ^**^P ≤ 0.01, and ^***^P ≤ 0.001).

To evaluate whether the 5′-3′ mRNA decay pathway is involved in ABA signaling we analyzed the impact of the depletion of major factors in decapping and exonucleolytic degradation, LSM1, DCP5 and XRN4, on typical ABA responses. First we investigated ABA sensitivity of the double *lsm1a lsm1b, dcp5-1*, and *xrn4-5* loss-of-function mutants (Souret et al., 2004; Xu and Chua, 2009; Golisz et al., 2013) during germination by determining average seed germination time (Pieper's index). As shown previously, under normal growth condition, *lsm1a lsm1b* and *dcp5-1* seeds germinated slightly later than the wild-type (the ratio of mutant vs. Col-0 Pieper's index > 1; Figure 1A) (Xu and Chua, 2009; Perea-Resa et al., 2012). For all concentrations of ABA, germination time of *dcp5-1* seedlings was significantly longer (higher Pieper's index) that the *dcp5-1* mutant is more susceptible to ABA. In turn, differences in germination time of *lsm1a lsm1b* plants were similar in the absence and in the presence of ABA and germination of *xrn4-5* was not altered at any conditions. Next, to assess ABA sensitivity of the mutants during early stages of vegetative growth, we measured primary root length in the presence of ABA. To this end 5-day-old seedlings grown in hormone-free medium were transferred to vertical plates with various concentrations of ABA (5 and 10 μM). The roots of *lsm1a lsm1b* and *xrn4-5* plants were clearly shorter than in the case of Col-0, whereas no effect was visible for the *dcp5-1* line (Figures 1B–G). These observations strongly suggest that these two mutants are hypersensitive to ABA during later stages of development. Together, the data indicates that DCP5, LSM1 and XRN4 are involved in response to ABA.

### DCP5 and LSM1-7 modulate mRNA stability and protein level of ABA receptors

**Figure 2:**
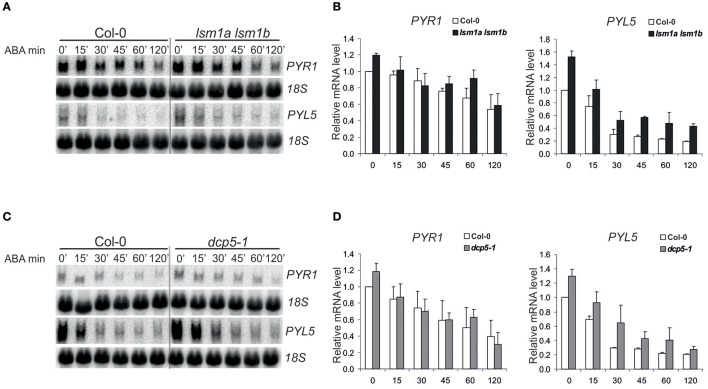
Expression of *PYR1* and *PYL5* in *lsm1a lsm1b*, and *dcp5-1* mutants following ABA treatment. Northern blot analysis of *PYR1* and *PYL5* mRNAs at specific time points following ABA treatment in 2-week-old *lsm1a lsm1b* **(A)** *and dcp5-1* **(C)** and Col-0 seedlings. Plants grown in hydroponic culture were treated with 50 μM of ABA for the indicated time. 18S rRNA was used as a loading control. Experiment was repeated at least two times with similar results. **(B,D)** Quantitation of mRNA level relative to 18S rRNA. Data, representing mean values of independent experiments, were calculated using ImageJ software and plotted after normalization to Col-0 at 0' time point.

**Figure 3:**
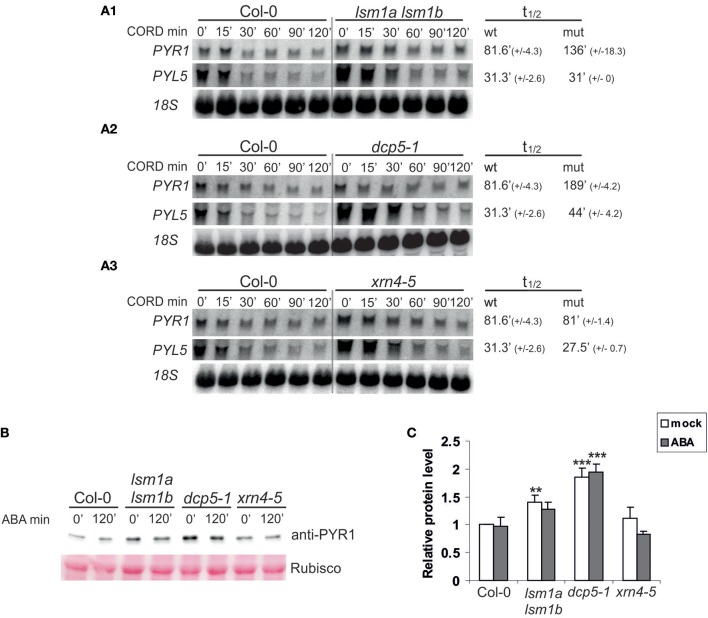
DCP5 and LSM1 modulate mRNA stability and protein level of ABA receptors. **(A)** Northern blot analysis of *PYR1* and *PYL5* mRNAs at specific time points after cordycepin treatment in 2-week-old *lsm1a lsm1b* **(A1)**, *dcp5-1* **(A2)**, *xrn4-5* **(A3)** mutants and Col-0. The estimated mRNA half-lives (t_1/2_) (mean values from at least two independent experiments) are shown to the right of each panel. 18S rRNA was used as loading control. Numbers in parentheses indicate SD. **(B)** Western blot analysis using anti-PYR1 antibodies for 2-week-old Col-0, *lsm1a lsm1b, dcp5-1*, and *xrn4-5* seedlings treated with 50 μM of ABA for the indicated time. Experiments were repeated three times with similar results. **(C)** Quantitation of PYR1 western blot chemiluminescence signals relative to the Ponceau S staining of Rubisco. Data from independent experiments were plotted and normalized to no-ABA Col-0 control. Error bars represent SD of three independent experiments. Asterisks indicate significant differences between Col-0 and the mutants (^**^P ≤ 0.01, ^***^P ≤ 0.001) for the indicated time point.

ABA receptors, RCAR/PYR/PYL proteins constitute a 14-member family and all of them, except PYL13, are able to activate ABA signaling (Fujii et al., 2009). PYL5, has been found among several proteins important for growth and development that are encoded by unstable transcripts (Gutierrez et al., 2002; Narsai et al., 2007). To check whether decapping regulates the level of PYL transcripts in response to ABA we analyzed mRNAs of *PYL5* and *PYR1* in 2-week-old Col-0 and decapping mutants *lsm1a lsm1b* and *dcp5-1* before and following ABA (50 μM) treatment. As expected, both transcripts are regulated by ABA and their level gradually decreases with time after the treatment (Figure 2). In *lsm1a lsm1b* and *dcp5-1* mutant lines *PYR1* level was slightly increased only in control conditions, while *PYL5* was visibly up-regulated before and during ABA treatment in *lsm1a lsm1b* and to a lesser extent in *dcp5-1*. To check whether the impact of LSM1 on ABA receptor mRNAs is direct we tested the stability of *PYL5* and *PYR1* mRNAs in the *lsm1a lsm1b* mutant following transcriptional inhibition by cordycepin. Northern blot analysis showed that half-life of *PYR1*, but not of *PYL5*, was markedly increased in the absence of LSM1 (Figure 3A1), suggesting that *PYR1* mRNA is a direct substrate of the LSM1-7 complex. This observation was confirmed for another mutant in the decapping complex, *dcp5-1*, where *PYR1* mRNA was clearly, and *PYL5* only marginally, stabilized (Figure 3A2). Surprisingly, half-lives of these transcripts were not altered in *xrn4-5* plants. These results indicate that at least one of PYL mRNAs, *PYR1*, is a direct substrate of DCP- and LSM-mediated decapping, but its turnover is independent of the cytoplasmic 5'-3' exoribonuclease XRN4. This is consistent with a finding that in *Arabidopsis* this nuclease is involved in the decay of only a subset of cellular mRNAs (Souret et al., 2004; Rymarquis et al., 2011; Golisz et al., 2013).

DCP5 has been reported to repress translation of *OLEO1* and *OLEO2* mRNAs encoding seed storage proteins (Xu and Chua, 2009). Since *PYR1* mRNA is a direct substrate of decapping complexes, we assumed that it might be translationally repressed, even if its level is not affected in plants lacking DCP5 and LSM1. To test this hypothesis we checked the level of PYR1 protein in the mutants using specific anti-PYR1 antibodies. We found that PYR1 strongly accumulated in *dcp5-1* and to a lower extent in *lsm1a lsm1b*, but not in *xrn4-5* plants (Figure 3B). These observation correlate well with extended half-life of *PYR1* mRNA in these mutants (Figures 3A–C).

### ABA-dependent SnRK2 kinases are modulated by 5′-3′ mRNA decay

**Figure 4:**
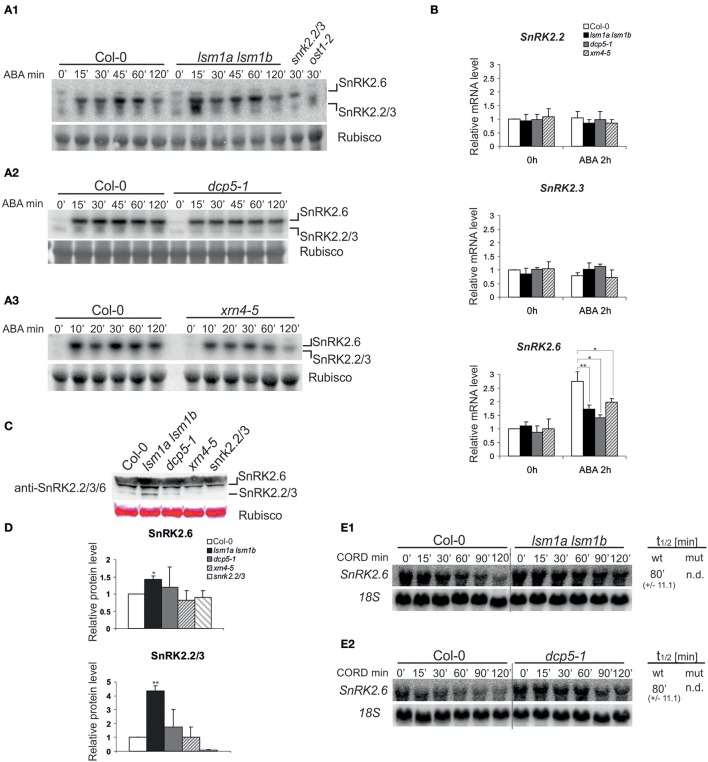
LSM1, DCP5, and XRN4 modulate the activity, protein level and mRNA stability, but not the steady state of ABA-dependent SnRK2 protein kinases **(A)** Protein kinase activity using protein extracts of 2-week-old Col-0, *lsm1a lsm1b, snrk2.2/3, ost1-2(snrk2.6)* **(A1)**, *dcp5-1* **(A2)** *and xrn4-5* **(A3)** plants. Extracts were prepared from plants grown in hydroponic cultures before and after treatment with 50 μM of ABA for the indicated time. Activity was monitored by the in-gel kinase activity assay with HIS3 as a substrate. Coomassie blue staining of Rubisco was used as a loading control. Migration of SnRK2.2/3 and SnRK2.6 kinases was assigned based on their absence in *snrk2.2/2.3* and *ost1-1/snrk2.6* mutants, SnRK2.2 and SnRK2.3 are not distinguishable due to the same molecular weight. Quantitation of the kinase activity is presented in Supplementary Figure 1. **(B)** RT-qPCR analysis of *SnRK2.2, SnRK2.3*, and *SnRK2.6* transcripts in 2-week-old Col-0, *lsm1a lsm1b, dcp5-1*, and *xrn4-5* plants grown in hydroponics culture exposed to 50 μM of ABA for 2 h. For each gene, the expression level is shown relative to Col-0 in control conditions set as 1. Error bars represent SD of three independent experiments. Asterisks indicate significant differences between Col-0 and the mutants (^*^P ≤ 0.05, ^**^P ≤ 0.01). **(C)** Western blot analysis using anti-SnRK2.2/3/6 antibodies for protein extracts prepared from 2-week-old Col-0, *lsm1a lsm1b, dcp5-1*, and *xrn4-5* seedlings. Ponceau S staining of Rubisco was used as a loading control. Experiment was repeated three times with similar results. To separate SnRK2.6 and SnRK2.2/3 the electrophoresis was performed in gradient gels (Invitrogen). **(D)** Quantitation of the chemiluminescence of SnRK2.2/3/6 western blot signals relative to the Ponceau S staining of Rubisco. Data from independent experiments were normalized to Col-0. Error bars represent SD of three independent experiments. Asterisks indicate significant differences between Col-0 and the mutants (^*^P ≤ 0.05, ^**^P ≤ 0.01). **(E)** Northern blot analysis of *SnRK2*.2 and *SnRK2*.6 mRNAs at specific time points after cordycepin treatment in 2-week-old *lsm1a lsm1b* **(E1)**, *dcp5-1* **(E2)** mutants and Col-0. The estimated mRNA half-lives (t_1/2_) (mean values from at least two independent experiments) are shown to the right of each panel. 18S rRNA was used as a loading control. Numbers in parentheses indicate SD.

The elevated level of PYR1 receptor in *lsm1a lsm1b* and *dcp5-1* mutants may increase ABA-dependent activation of SnRK2.2/3/6 (Fujii et al., 2009). We therefore measured the activity of SnRK2 kinases in *lsm1a lsm1b, dcp5-1* and *xrn4-5* mutants *in vivo* before and after exposure to ABA (50 μM) by an in-gel kinase assay using *Arabidopsis* crude protein extracts and histone H3 as a substrate (Yoshida et al., 2002). Extracts prepared from Col-0, *snrk2.2/3* and *ost1/snrk2.6* seedlings were used as controls. Consistent with previous reports, ABA treatment caused rapid activation of SnRK2.2/3/6 protein kinases (Figure 4A). Activity of SnRK2.6 and SnRK2.2/3 in *lsm1a lsm1b* mutant, but not in *dcp5-1* or *xrn4-5*, was stronger than in Col-0 at early time points (15 and 30 min, depending on the experiment, see (Supplementary Figure 1), reaching later the same level as in control plants.

It appears that SnRK2 activity corresponds to changes in PYR1/PYL5 receptors only in *lsm1a lsm1b* plants, therefore we conclude that SnRK2 kinases are not generally regulated by the decapping/5′-3′ mRNA decay pathway. As increased activity of SnRK2.2/3/6 in *lsm1a lsm1b* might be caused by higher amount of kinases we tested their mRNA and protein level in the three mutants under study. Real-time quantitative PCR (RT-qPCR) analysis of *SnRK2.2/3/6* mRNAs revealed no differences in the case of *SnRK2.2* and *SnRK2.3* between control and the mutants (Figure 4B), but as reported previously *SnRK2.6* mRNA was strongly induced after ABA treatment in Col-0 plants (Chan, 2012), and only moderately in all mutants. In turn, using western blotting with anti- SnRK2.2/3/6 antibodies we observed a more intense signal corresponding to SnRK2.2/2.3 and SnRK2.6 kinases in *lsm1a lsm1b*, but not in *dcp5-1* and *xrn4-5* (Figure 4C). This result suggests that increased activity of ABA-dependent SnRK2s in the *lsm1a lsm1b* mutant is due to higher protein level of these kinases. Considering that LSM1-7 and DCP5 may be involved in translation repression we also tested the stability of *SnRK2* mRNAs in *lsm1a lsm1b* and *dcp5-1* mutants following transcriptional inhibition by cordycepin. Since *SnRK2.2* and *SnRK2.3* have particularly long half-lives (>120 min, data not shown, Narsai et al., 2007) we were able to determine only the half-life of *SnRK2.6*. This transcript was strongly stabilized in the *lsm1a lsm1b* mutant and to a lesser extent in the *dcp5-1* line (Figure 4D). Higher stability of *SnRK2.6* in *lsm1a lsm1b* plants correlates well with the increased level of the protein and suggests that at least *SnRK2.6* mRNA is affected by decapping complex and its translation is repressed by the LSM1-7 complex.

### The *Arabidopsis* LSM1-7 complex, DCP5 and XRN4 contribute to the regulation of gene expression in response to ABA

**Figure 5:**
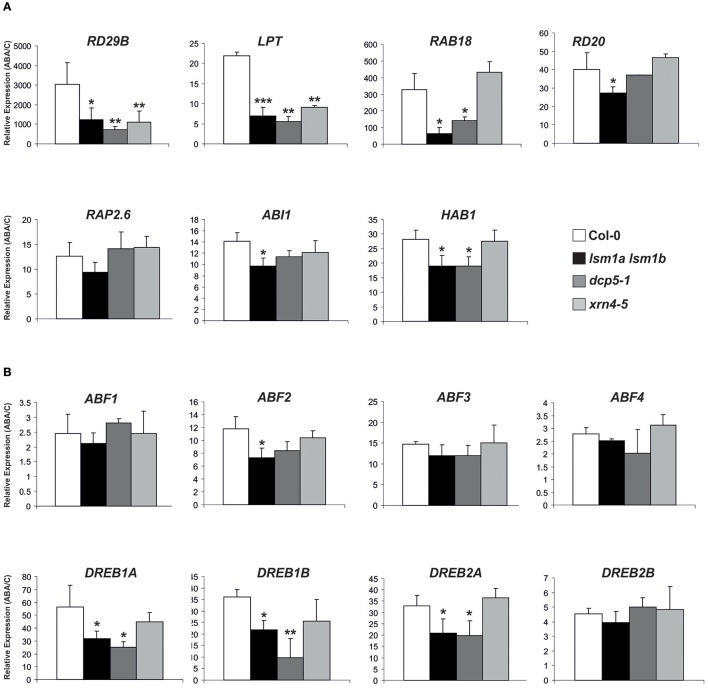
LSM1 and DCP5 indirectly modulate the expression of ABA-responsive genes. Expression of selected ABA-inducible genes **(A)** and *ABFs* and *DREBs* transcription factors **(B)** determined by RT-qPCR in 2-week-old Col-0, *lsm1a lsm1b, dcp5-1*, and *xrn4-5* plants grown in hydroponic culture exposed to 50 μM of ABA for 2 h. Values are expressed relative to Col-0 set as 1. Error bars represent SD of three independent experiments. Asterisks indicate significant differences between Col-0 and the mutants (^*^P ≤ 0.05, ^**^P ≤ 0.01, and ^***^P ≤ 0.001).

To gain insight into the functional relationship between decapping/5′-3′ mRNA decay and the PYL/PYR/RCAR-PP2C-SnRK2 signaling pathway we examined the induction of ABA- and SnRK2-inducible transcripts, including *RD29B, RAB18, RD20, LTP* (*At4g33550*), *RAP2.6, ABI1*, and *HAB1* (Fujita et al., 2009). As reported previously *RAB18, LPT, RD20, ABI1, HAB1* were up-regulated in *lsm1a lsm1b* plants in control conditions (Golisz et al., 2013; Supplementary Figures 2, 3), while only *LPT* accumulated in *dcp5-1* and *HAB1* in *xrn4-5* (Supplementary Figure 2). In contrast, *RAB18* and *RD20* mRNAs were significantly decreased in plants lacking XRN4. After 2 h exposure to ABA all examined transcripts were induced in Col-0 plants as expected, but this effect was much reduced in ABA-treated *lsm1a lsm1b* plants for almost all tested mRNAs, except *RAP2.6* (Figure 5A). Similarly, the expression of *RD29B, LTP, RAB18*, and *HAB1* after exposure to ABA was lower in *dcp5-1* compared to Col-0, while only induction of *RD29B* and *LTP* was decreased in *xrn4-5* plants.

Since higher ABA-induced activation of SnRK2 in *lsm1a lsm1b* seedlings occurs within 15–30 min and then reaches the same level as in Col-0, we checked whether the expression pattern of ABA-inducible genes exhibits any fluctuations in the mutant. Northern blot analysis of chosen transcripts at different time points after ABA treatment showed that all tested mRNAs are evenly induced, alike in control and mutant plants (Supplementary Figure 3). These results suggest that decapping-mediated accumulation of common ABA-responsive genes is independent of the activation of PYL receptors and SnRK2 kinases.

Recently it has been reported that SnRK2 kinases together with ABF2, ABF3, and ABF4 transcription factors may act as key regulators in mediating ABA-triggered chlorophyll (Chl) degradation and leaf senescence in Arabidopsis (Gao et al., 2016). To check whether the decapping complex contributes to the regulation of this pathway during subsequent vegetative growth stages we measured Chl in 3-week old plants after ABA treatment. Consistent with previous data chlorophyll level in plants treated with ABA was markedly reduced, but we did not observe differences in chlorophyll degradation between control and mutant plants (Supplementary Figure 4).

The PYL/PYR/RCAR-PP2C-SnRK2 pathway regulates gene expression through the phosphorylation of ABF transcription factors that cooperate with DREB factors (reviewed in Joshi et al., 2016). We therefore analyzed by RT-qPCR the mRNA levels of eight ABA-responsive transcription factors (four ABFs and four DREBs) involved in PYL/PYR/RCAR-PP2C-SnRK2 signaling during vegetative growth stage (Yoshida et al., 2015). Their level was either moderately altered or unaffected in *lsm1a lsm1b, dcp5-1*, and *xrn4-5* plants in control conditions without ABA treatment (Supplementary Figure 2) with little similar tendencies between the mutants, except that mRNAs of all four tested DREBs were down-regulated in *dcp5-1* and *ABF1* was significantly decreased both in *lsm1a lsm1b* and *dcp5-1*. Interestingly, this analysis revealed induction of all tested TF mRNAs in response to ABA and showed that activation of *DREB1A, DREB1B* and *DREB2A* was markedly reduced in *lsm1a lsm1b* and *dcp5-1* lines, whereas induction of *ABF2* was lower only in *lsm1a lsm1b* plants (Figure 5B). These findings suggest that DCP5 and the LSM1-7 complex modulate, probably indirectly, the expression of TFs involved in the PYL/PYR/RCAR-PP2C-SnRK2 pathway.

## Discussion

Abscisic acid is a key plant hormone involved in development and stress response. The past decade revealed a complex signal transduction network leading to transcriptomic, proteomic and metabolic reprogramming induced by ABA (Finkelstein, 2013). The best-known PYL/PYR/RCAR-PP2C-SnRK2 signaling pathway is responsible for regulating different biological processes, including regulation of transcription through ABFs/AREBs and DREBs transcription factors (reviewed in Yang et al., 2017). Growing evidence suggests the existence of a strong connection between RNA metabolism, abiotic stress and ABA signaling (reviewed in Kawa and Testerink, 2016). The altered ABA sensitivity of *A. thaliana* mutants with defects in RNA quality control factors implies possible involvement of post-transcriptional processes in the plant response to this hormone (Xiong et al., 2001; Zhang et al., 2011; reviewed in Hirayama and Shinozaki, 2007).

Here, we show that components of the cytoplasmic 5′-3′ mRNA decay pathway, DCP5, LSM1-7, and XRN4, contribute to ABA signaling in Arabidopsis. Our analysis of mutants in these factors shows that, although they are all hypersensitive to ABA, physiological and molecular effects are not the same. Upon ABA treatment germination is more severely inhibited in plants lacking DCP5, whereas later developmental stages, i.e., primary root growth, are strongly affected in *lsm1a lsm1b* and *xrn4-5* mutants. We observed that DCP5 and LSM1 regulate mRNA stability of the core factors of early ABA signaling, ABA-receptor PYR1 and SnRK2 kinase, and LSM1 indirectly affects the level of *PYL5* mRNA. In turn, the amount of PYR1 and SnRK2s proteins depends on DCP5 and LSM1 but not XRN4. Increased expression of these proteins without changes in the steady state level of corresponding mRNAs strongly suggests that LSM1-7 and DCP5, possibly as a part of the decapping complex, are involved in translational repression of the main components of ABA signaling. Recent findings show that depending on stress conditions the LSM1-7 complex binds a different set of stress-inducible transcripts, targeting them for decapping and subsequent degradation (Perea-Resa et al., 2016). It is therefore possible that observed miscorrelations of stress-induced changes in Arabidopsis transcriptome and proteome can be explained by the interplay between selective degradation and translational repression of different substrates (Kawaguchi et al., 2004; Jiang et al., 2007).

Analyzed mutants also show varying levels of ABA-dependent SnRK2s kinase activity, increased in *lsm1a lsm1b* plants and decreased in *dcp5-1* and *xrn4-5* when compared to the wild-type. These findings, however, do not explain reduced expression of ABA-responsive genes in *lsm1a lsm1, dcp5-1* mutants and to a lesser degree in *xrn4-5*. This probably arises from the complexity of ABA signaling. Our results suggest that positive regulation of known ABA- and SnRK2-inducible genes, such as *RD29B* and *RAB18*, by DCP and LSM1-7 complex is at least partially due to indirect modulation of expression of transcription factors, including *ABFs* and *DREBs*. These findings are consistent with a study showing that mRNA decapping is involved directly and indirectly in dehydration stress response in *Arabidopsis* via regulation of *DREB* transcription factors (Xu and Chua, 2012).

It has been shown recently that PAP (3′-phosphoadenosine 5′-phosphate), the inhibitor of XRNs exoribonucleases, in concert with nuclear XRN2 and XRN3 participates in ABA signaling pathway alternative to the canonical PYL/PYR/RCAR-PP2C-SnRK2 (Pornsiriwong et al., 2017). PAP-XRN2/3 up-regulates the expression of multiple ABA signaling components, especially Calcium Dependent Protein Kinases (CDPKs) and Calcineurin B-Like Protein-Interacting protein kinases (CIPKs), which activate transcription of downstream ABA signaling genes (reviewed in Yu et al., 2013; Boudsocq and Sheen, 2014). However, the expression of CDPKs and CIPKs was not altered either in *lsm1a lsm1b* or *xrn4-5* (Estavillo et al., 2011; Golisz et al., 2013), suggesting that components of the cytoplasmic 5′-3′ RNA decay modulate ABA-mediated transcriptional regulation in a different manner than the PAP-XRN2/3 pathway. Although existence of the cytoplasmic PAP-XRN4 pathway has not been confirmed (Estavillo et al., 2011), a potential ABA-induced inhibition of XRN4 by PAP may explain different molecular phenotypes between decapping mutants and *xrn4-5*. Considering the PYL/PYR/RCAR-PP2C-SnRK2-independent function of PAP-XRN2/3 in mediating stomata closure in guard cells (Pornsiriwong et al., 2017), it is tempting to speculate that the cytoplasmic 5′-3′ mRNA decay factors, DCP, LSM1-7 and XRN4, may also contribute to the regulation of other processes than gene expression in ABA signaling, for example stomata closure.

Together, our findings show that DCP5 and LSM1 regulate mRNA stability and act in translational repression of the main components of the early ABA signaling, PYR/PYL ABA receptors and SnRK2s protein kinases. DCP and LSM1-7 complexes also appear to modulate ABA-dependent expression of stress related transcription factors from the AP2/ERF/DREB family that in turn affect the level of genes regulated by the PYL/PYR/RCAR-PP2C-SnRK2 pathway. Taken into consideration that decapping factors DCP1 and VCS become phosphorylated in response to water stress and are regulated by upstream MPK6 and ABA-independent SnRK2s kinases, respectively (Xu and Chua, 2012; Soma et al., 2017), we can speculate that the action of the decapping complex during abiotic stress may be governed by different mechanisms in ABA-dependent and independent pathways.

## Author contributions

IW performed qRT-PCRs, immunoblots, in-gel kinase assays in Figures 3–5, Supplementary Figures 1, 2, Northern blots in Supplementary Figure 3 and contributed to Figure 2. AG and DK performed phenotyping analysis in Figure 1. AG performed transcripts stability assays in Figures 3, 4. AS contributed to Supplementary Figure 2 and Figure 5, and AK to Figure 4. IW generated all figures. JK and IW designed the project and wrote the manuscript.

### Conflict of interest statement

The authors declare that the research was conducted in the absence of any commercial or financial relationships that could be construed as a potential conflict of interest.

## Supplementary material

Supplementary Figure 1 LSM1 modulates the activity of ABA-dependent SnRK2 protein kinases—supplement to Figure 4. **(A)** Quantitation of SnRK2.2/3/6 activity (radioactive signals) from blots in Figure 4A relative to the Coomassie blue staining of Rubisco. Data were plotted and normalized to Col-0 using ImageJ software. **(B)** Protein kinase activity using protein extracts from 2-week-old Col-0 and *lsm1a lsm1b* plants grown in hydroponic cultures before and after treatment with 50 μM of ABA for the indicated times. Activity was monitored by the in-gel kinase activity assay with HIS3 as a substrate. Coomassie blue staining of Rubisco was used as a loading control. Migration of SnRK2.2 and SnRK2.3 are not distinguishable due to the same molecular weight. **(C)** Quantitation of SnRK2.2/3/6 activity (radioactive signals) from blots in **(B)** relative to the Coomassie blue staining of Rubisco. Data were plotted and normalized to Col-0 using ImageJ software.

Supplementary Figure 2 Expression levels of ABA-inducible genes in Col-0, *lsm1a lsm1b, dcp5-1*, and *xrn4-5* plants. RT-qPCR analysis of the expression of selected ABA- and SnRK2-inducible genes **(A)** and *ABFs* and *DREBs* transcription factors **(B)** in *lsm1a lsm1b, dcp5-1*, and *xrn4-5* plants grown in hydroponic culture. Values are expressed relative to Col-0 set as 1. Error bars represent SD of three independent experiments. Asterisks indicate significant differences between Col-0 and the mutants (^*^*P* ≤ 0.05, ^**^*P* ≤ 0.01, and ^***^*P* ≤ 0.001).

Supplementary Figure 3 Expression of ABA-inducible genes in *lsm1a lsm1b* following ABA treatments Northern blot analysis of selected ABA-inducible genes in 2-week-old *lsm1a lsm1b* and Col-0 plants grown in hydroponic culture treated with 50 μM of ABA for the indicated time. 18S rRNA was used as a loading control.

Supplementary Figure 4 Phenotypic characterization of Col-0, *lsm1a lsm1b, dcp5-1*, and *xrn4-5* leaves after ABA treatment. **(A)** Detached leaf discs from 3-week-old plants were treated with water (mock) or 15 μM ABA for 2 days. Pictures represent one of three replicas. **(B)** Chl degradation in leaves shown in **(A)** was measured as relative Chl content after ABA treatment to control conditions. Data are means ± SD of three replicates. Experiment was repeated two times with similar results.

Supplementary Table 1 List of Primers.
