# Supplementary figures and images for: mRNA Decapping and 5′-3′ Decay Contribute to the Regulation of ABA Signaling in Arabidopsis thaliana
Source: Front Plant Sci. 2018 Mar 12;9:312. doi: 10.3389/fpls.2018.00312 (PMC5857609; doi:10.3389/fpls.2018.00312)

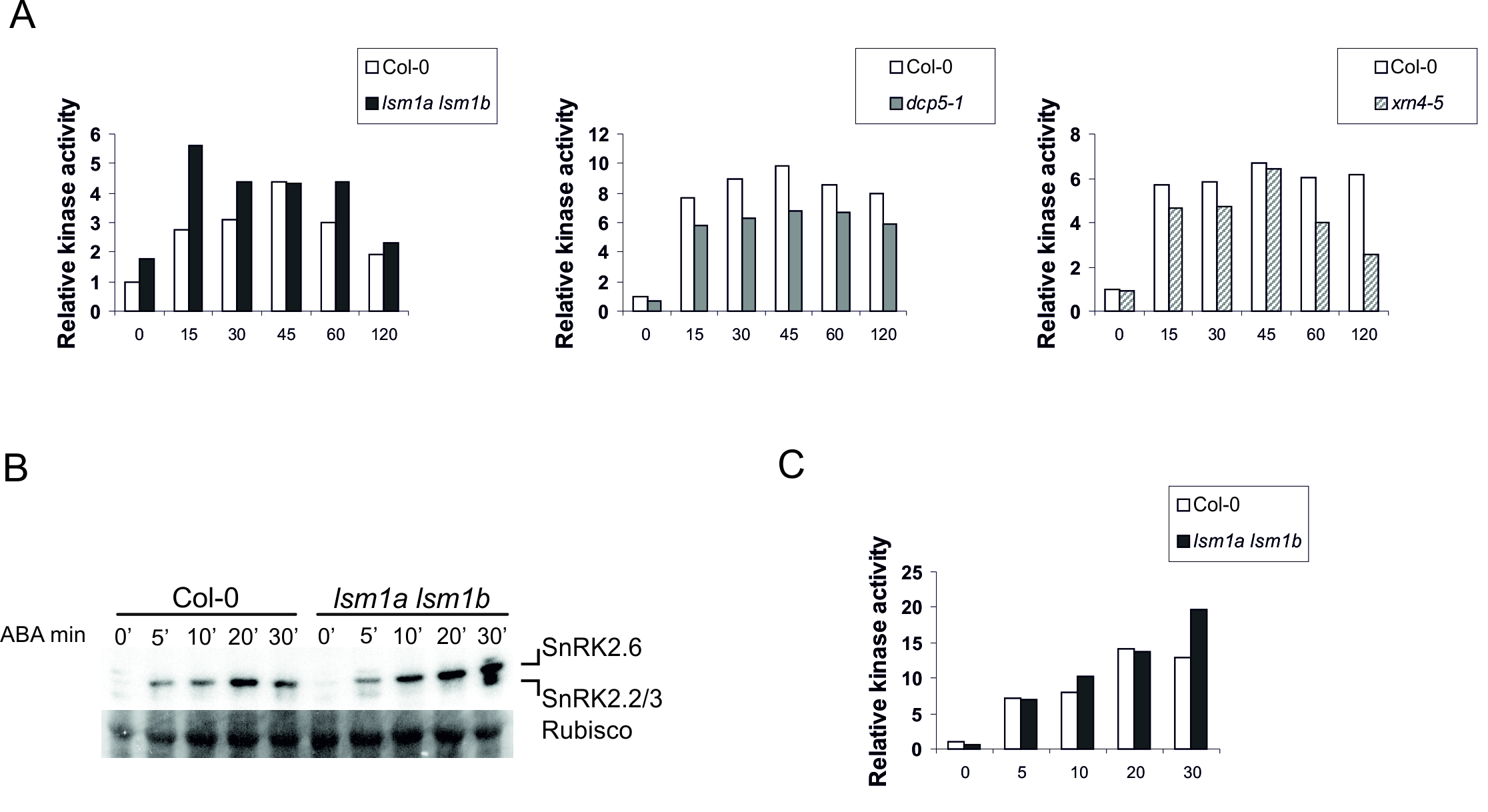

Supplement: Supplementary Figure 1 — LSM1 modulates the activity of ABA-dependent SnRK2 protein kinases—supplement to Figure 4. (A) Quantitation of SnRK2.2/3/6 activity (radioactive signals) from blots in Figure 4A relative to the Coomassie blue staining of Rubisco. Data were plotted and normalized to Col-0 using ImageJ software. (B) Protein kinase activity using protein extracts from 2-week-old Col-0 and lsm1a lsm1b plants grown in hydroponic cultures before and after treatment with 50 μM of ABA for the indicated times. Activity was monitored by the in-gel kinase activity assay with HIS3 as a substrate. Coomassie blue staining of Rubisco was used as a loading control. Migration of SnRK2.2 and SnRK2.3 are not distinguishable due to the same molecular weight. (C) Quantitation of SnRK2.2/3/6 activity (radioactive signals) from blots in (B) relative to the Coomassie blue staining of Rubisco. Data were plotted and normalized to Col-0 using ImageJ software. [file Image1.JPEG]

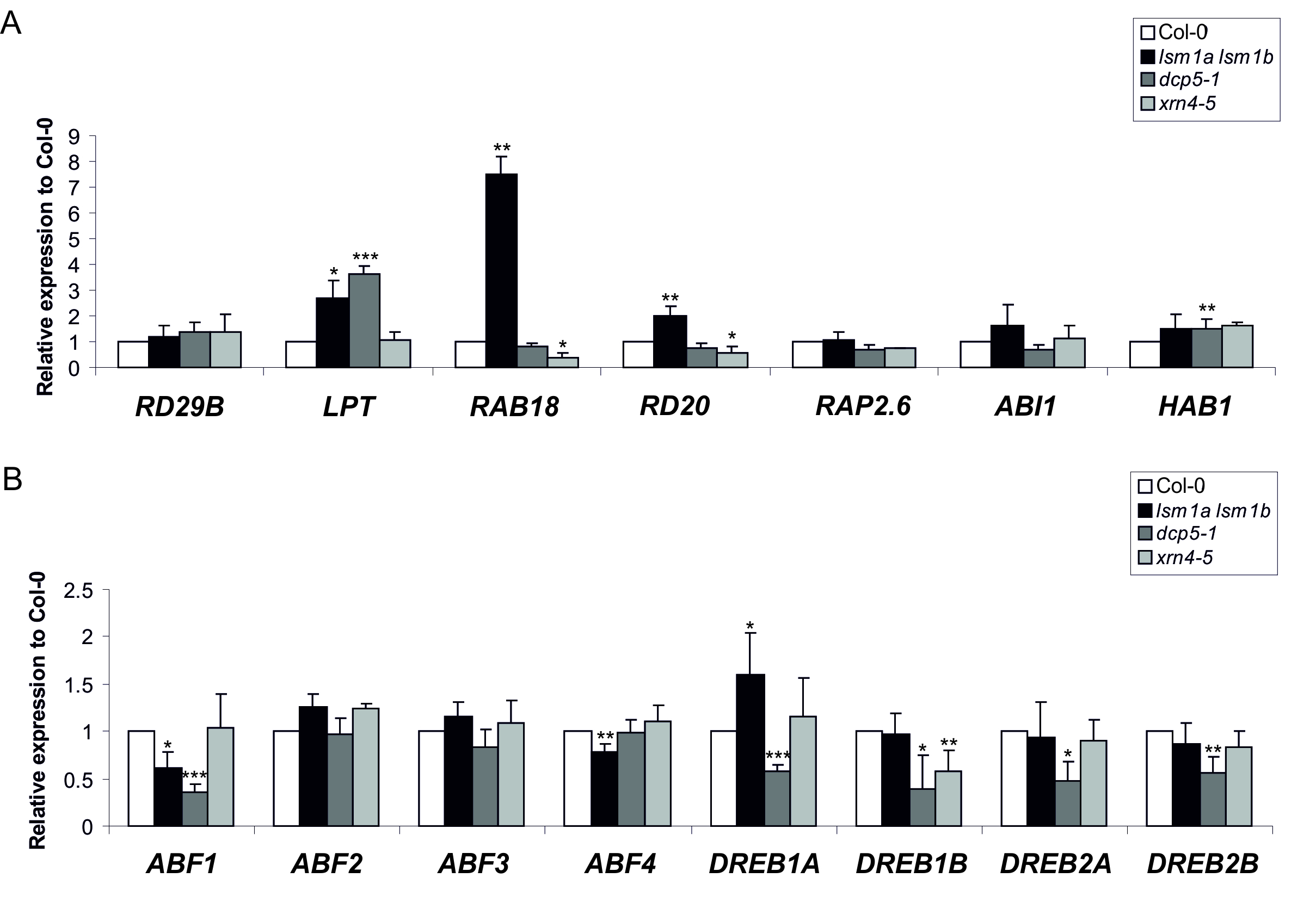

Supplement: Supplementary Figure 2 — Expression levels of ABA-inducible genes in Col-0, lsm1a lsm1b, dcp5-1, and xrn4-5 plants. RT-qPCR analysis of the expression of selected ABA- and SnRK2-inducible genes (A) and ABFs and DREBs transcription factors (B) in lsm1a lsm1b, dcp5-1, and xrn4-5 plants grown in hydroponic culture. Values are expressed relative to Col-0 set as 1. Error bars represent SD of three independent experiments. Asterisks indicate significant differences between Col-0 and the mutants (*P ≤ 0.05, **P ≤ 0.01, and ***P ≤ 0.001). [file Image2.jpg]

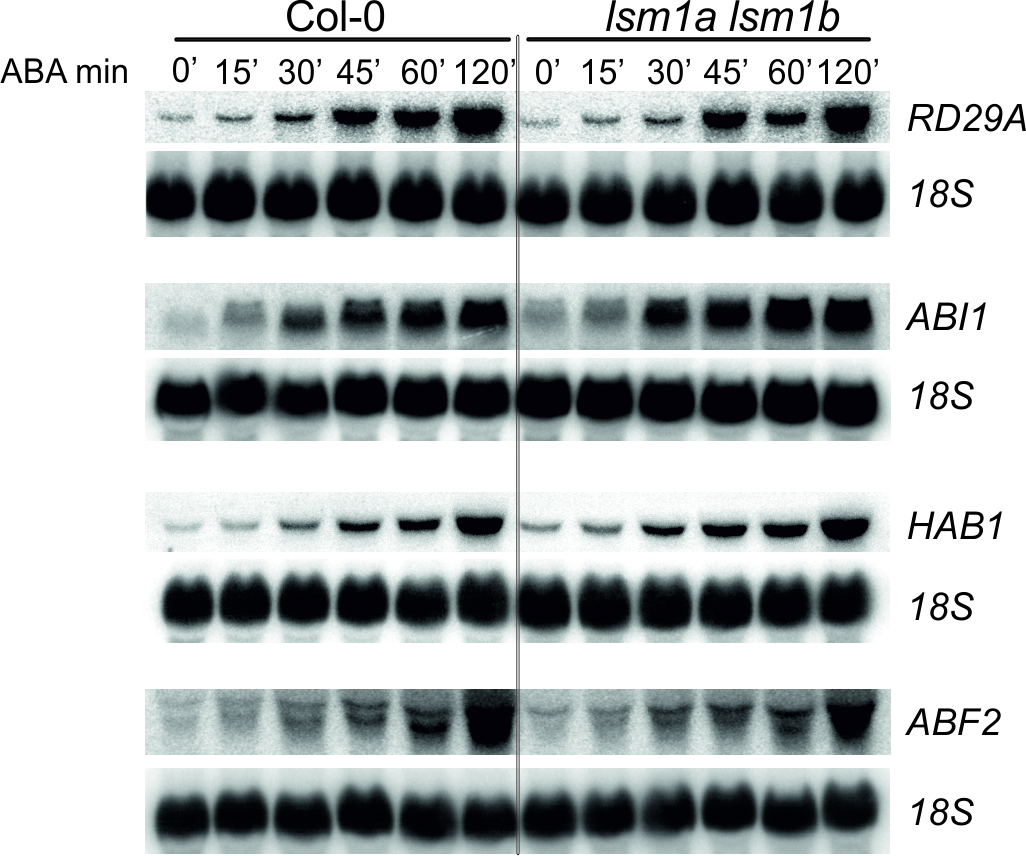

Supplement: Supplementary Figure 3 — Expression of ABA-inducible genes in lsm1a lsm1b following ABA treatments Northern blot analysis of selected ABA-inducible genes in 2-week-old lsm1a lsm1b and Col-0 plants grown in hydroponic culture treated with 50 μM of ABA for the indicated time. 18S rRNA was used as a loading control. [file Image3.JPEG]

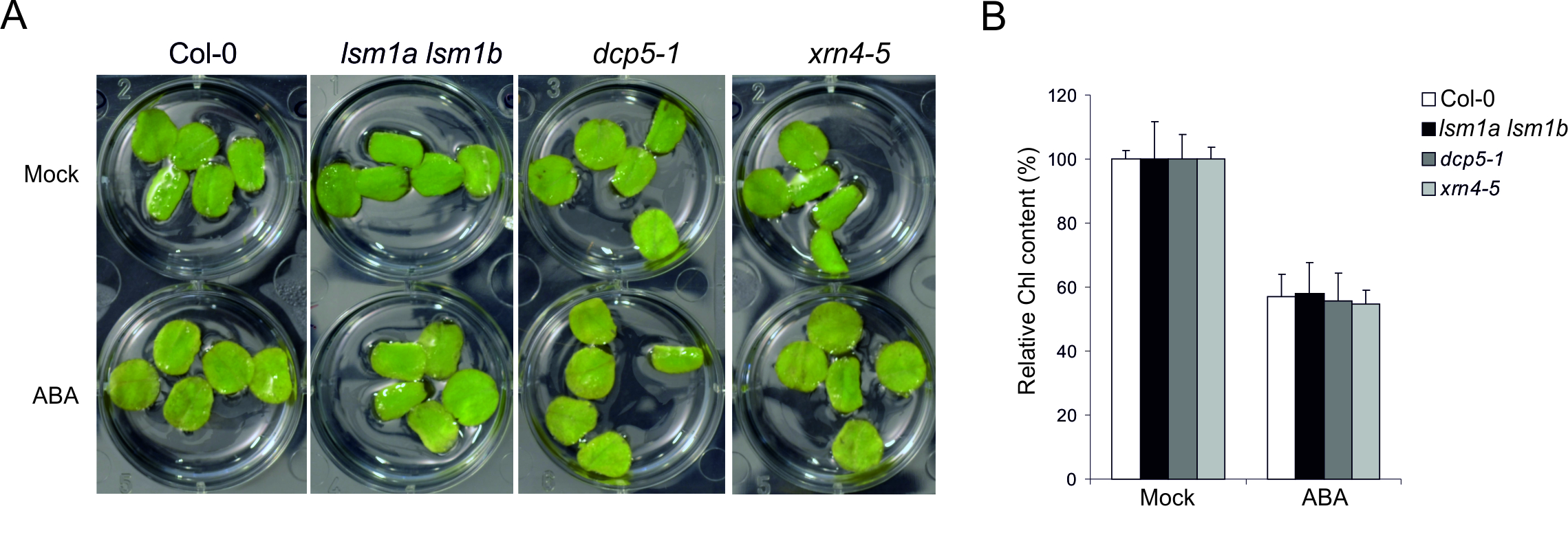

Supplement: Supplementary Figure 4 — Phenotypic characterization of Col-0, lsm1a lsm1b, dcp5-1, and xrn4-5 leaves after ABA treatment. (A) Detached leaf discs from 3-week-old plants were treated with water (mock) or 15 μM ABA for 2 days. Pictures represent one of three replicas. (B) Chl degradation in leaves shown in (A) was measured as relative Chl content after ABA treatment to control conditions. Data are means ± SD of three replicates. Experiment was repeated two times with similar results. [file Image4.JPEG]
